# Predictors of the onset of neonatal sepsis at the Neonatal Intensive Care Unit of a tertiary hospital in Ghana: A cross‐sectional study

**DOI:** 10.1002/hsr2.1673

**Published:** 2023-11-02

**Authors:** Kwame Opare‐Asamoah, Samuel E. Acquah, Ezekial Kofi Vicar, Lawrence Quaye, Abdul‐Mumin Alhassan, Saeed F. Majeed, Vida Nyagre Yakong, Samuel Yankson

**Affiliations:** ^1^ Department of Biological Sciences, Faculty of Biosciences University for Development Studies Tamale Ghana; ^2^ Department of Clinical Microbiology, School of Allied Health Sciences University for Development Studies Tamale Ghana; ^3^ Department of Clinical Microbiology, School of Medicine University for Development Studies Tamale Ghana; ^4^ Department of Biomedical Laboratory Sciences, School of Allied Health Sciences University for Development Studies Tamale Ghana; ^5^ Department of Pediatrics and Child Health, School of Medicine University for Development Studies Tamale Ghana; ^6^ Department of Preventive Health Nursing, School of Nursing and Midwifery University for Development Studies Tamale Ghana; ^7^ Department of Physiology and Biophysics, School of Medicine University for Development Studies Tamale Ghana

**Keywords:** early‐onset sepsis, late‐onset sepsis, neonates, NICU, predictors, X teaching hospital

## Abstract

**Background and Aim:**

Neonatal sepsis is a systemic inflammatory response to infection during the first 4 weeks of an infant's life. It is a significant cause of neonatal morbidity and mortality in low‐ and middle‐income countries. This study aimed to determine the predictors of the onset of sepsis at the Neonatal Intensive Care Unit of the Tamale Teaching Hospital, Ghana.

**Methods:**

A cross‐sectional study was conducted among 275 mothers and their singleton neonates diagnosed clinically with sepsis. A univariate and multivariate logistic regression analysis adjusted for maternal occupational status was performed to determine the maternal and neonatal predictors of early‐onset (EOS) and late‐onset sepsis (LOS), respectively.

**Results:**

Single motherhood (AOR = 1.882, 95% CI = 0.926−3.822, *p* = .08) and home delivery (AOR = 3.667, 95% CI = 0.584−23.026, *p* = .17) were predictors of EOS, with single motherhood being the predictor for LOS (AOR = 2.906, 95% CI = 0.715−11.805, *p* = .14) in a univariate analysis. When maternal occupation was adjusted for in a multivariate analysis, single mother (AOR = 2.167, 95% CI = 1.010−4.648, *p* = .04) was the main predictor of EOS, with low neonatal birth weight being the main predictor of LOS (AOR = 0.193, 95% CI = 0.038−0.971, *p* = .04).

**Conclusion:**

Maternal marital status is a significant predictor of both EOS and LOS, with predictors of EOS being lower gestational age and low birth weight, while for LOS, low birth weight is the main predictor. Findings from this study can serve as a commencement point for developing predictive models for the onset of sepsis in neonates in the study facility.

## Key points

What is known: Neonatal sepsis is a medical emergency.

What is new: Predictors for the identification of early and late‐onset sepsis in a resource‐poor setting have been identified in this study.

The clinical implications of this study: Findings from this provide a valuable reference for the early identification of neonatal sepsis to improve clinical outcomes. Also, the findings in this study will serve as a commencement point for developing predictive models for identifying the onset of sepsis in neonates in resource‐poor settings in Sub‐Saharan Africa.

## 1 INTRODUCTION

Neonatal sepsis is the systemic inflammatory response to bacterial infection during the first 28 days of an infant's life. Neonatal sepsis is a significant contributor to neonatal morbidity and mortality and according to a World Health Organization (WHO) report, it accounts for 15% of the 2.5 million neonatal deaths worldwide, 98% of which occur in developing nations like Ghana.^1^ Neonatal sepsis is of significant public health importance in Sub‐Saharan Africa as it poses a serious drawback to the progress made in health care delivery systems in these less‐resourced countries.^2^, ^3^, ^4^

Reports from many Sub‐Saharan African countries indicate varying prevalence and factors regarding neonatal sepsis.^2^ However, notable factors associated with neonatal sepsis‐related morbidity and mortality may include unhygienic delivery places, unsafe delivery, inappropriate cord care practices, and inadequate or lack of antenatal.^2^, ^5^, ^6^ Additionally, inadequate or lack of antenatal care may lead to failure or delay in identifying newborns at risk of neonatal sepsis for immediate clinical attention.^4^ Similarly, home deliveries that are poorly supervised or unsupervised, prematurity with its associated low birth weight, and failure to practise exclusive breastfeeding have been identified as risk factors associated with neonatal sepsis.^7^ Institutionally, poor infrastructure, health systems failure, inadequate logistics, poor maternal and child welfare intervention coverage among low‐income earners, and weak economies underpin the increase in infection rates and sepsis‐related deaths.^8^

Neonatal sepsis is broadly classified into two categories based on onset time. Early‐onset sepsis (EOS) when the sepsis occurs within the first 7 days of life, and late‐onset sepsis (LOS) if it occurs after 7 days postnatally.^9^ The case severity of sepsis tends to vary between developing and developed countries. In developed countries, EOS is more severe, with a higher case mortality rate, while LOS is mostly associated with lower morbidity and mortality in the same settings.^10^ However, in developing countries, case fatality for LOS has been reported to be higher than EOS.^11^

In terms of causes associated with the onset of sepsis, conditions such as premature membrane rupture, maternal urinary tract infection, and meconium‐stained amniotic fluid have been associated with EOS's aetiology.^12^ While, the failure of early breastfeeding initiation, prolonged parenteral nutrition, hospitalization, surgery, long‐term use of invasive procedures such as intravascular catheterization and mechanical ventilation, and underlying cardiovascular and respiratory diseases had been reported as possible risk factors in the etiology of LOS.^13^

Neonatal sepsis presents with nonspecific clinical signs and symptoms, and it is regarded as a medical emergency necessitating empirical treatment with or without laboratory confirmation in resource‐limited settings.^14^ Such empirical treatment inadvertently exposes these neonates to antibiotics that otherwise may not be needed in some circumstances, contributing to antimicrobial resistance.^15^ Identifying neonates at a higher risk of sepsis and providing early intervention is one of the primary keys to reducing child morbidity and mortality to achieve Goal 3 of the Sustainable Development Goals.^16^ This study, therefore, determined the maternal and neonatal predictors of the onset of sepsis among neonates admitted to the Neonatal Intensive Care Unit (NICU) of the Tamale Teaching Hospital (TTH), Ghana.

## 2 METHODS

### 2.1 Study area

The study was conducted at the NICU of the TTH, Ghana. Tamale is the largest city and the only metropolitan area in northern Ghana. The TTH is the only tertiary referral hospital in northern Ghana and serves as a training center for health care institutions in its catchment area. The TTH has eight clinical Departments, including the Pediatrics and Child Health Department, of which the NICU is a subunit. The NICU is a 50‐crib/incubator capacity and a seven‐bed Kangaroo Mother unit with one consultant pediatrician, two medical officers, three pediatric nurses and 37 general nurses, and it provides advanced and specialized health care to neonates.^17^

### 2.2 Study population and design

This cross‐sectional study was conducted from January 2020 to June 2021 among mothers and their neonates (≤28 days) admitted to the NICU. All neonates who were clinically diagnosed as having sepsis by the attending clinician using the WHO guidelines for sepsis screening and in collaboration with the definition of septicaemia by the International Pediatric Sepsis Conference^18^, ^19^ as part of the established protocol at the facility were recruited into the study. Mothers/neonates without complete information in their medical records books were excluded from the study.

### 2.3 Sampling technique and sample size determination

A purposive sampling technique was employed to select mothers and their neonates for the study.^18^, ^19^ Cochran's formula^20^ and an estimated septicaemia prevalence of 21.9%, as Labi, Obeng‐Nkrumah^21^ reported among neonates in a tertiary health facility in Ghana, were used to estimate the minimum sample size of 262, of which 10% was added for attrition.

### 2.4 Data collection tool

A self‐designed data collection tool was used to collect maternal sociodemographic data such as age, marital status, educational status, and occupation. Neonatal clinical records such as the onset of sepsis, age on admission, birth weight, gestational age, duration of hospitalization, the axillary temperature on admission and place of delivery were extracted as recorded in the medical records books of mothers/neonates. Neonatal birth weight was classified as low birth weight (<2.5 kg), normal birth weight (2.5−4.0 kg), and high birth weight (>4.0 kg).^22^ The gestational age of the neonates was categorized as preterm (delivered < 37 completed weeks of gestation) and term (delivered ≥ 37 completed weeks of gestation).^22^ The axillary temperature of neonates on admission was defined as hypothermia (<36.5°C), normothermia (36.5°C−37.5°C), and hyperthermia (>37.5°C).^23^ The place of delivery was categorized as home if delivery occurred in the house, primary if delivery happened in a primary health center, secondary if delivered at a secondary health facility and tertiary if the birth occurred in a tertiary health facility. Neonatal sepsis was defined as EOS if it occurred within the first 7 days of life and LOS if it occurred 7 days after birth.^24^

### 2.5 Data analysis

All complete data were entered into Statistical Package for the Social Science (SPSS) v23 (IBM Corp, USA) and Microsoft Office Excel, 2019 (Microsoft, USA) for data analysis. Continuous variables were presented as means ± standard deviation and categorical variables were presented as frequencies and percentages. Student *t*‐test and *χ* ^2^ were used to test for association between study variables where appropriate. A univariate and multivariate logistic regression analysis adjusted for maternal occupational status was performed to determine the maternal and neonatal predictors of early and LOS among neonates. A *p*‐value of <.05 was set as the significance level for all statistical tests.

## 3 RESULTS

### 3.1 Maternal sociodemographic characteristics

In all, 275 mothers with a mean age of 26.5 ± 4.3 years and their singleton neonates participated in the study. Sixty‐eight percent (187/275) of the mothers were within the age group of 20−29 years, 26.2% (72/275) were aged > 29 years and 5.8% (16/275) were aged < 20 years. Most (82.5% [227]) of these mothers were married, with 45.8% (126) having no formal education and 57.8% (159) being self‐employed (Table 1).

**Table 1:** Maternal sociodemographic characteristics.

Variable	*n* (%)
Age (years)	
<20	16 (5.8)
20−29	187 (68.0)
>29	72 (26.2)
Marital status	
Single	48 (17.5)
Married	227 (82.5)
Educational status	
None	126 (45.8)
Basic	82 (29.8)
Secondary	55 (20.0)
Tertiary	12 (4.4)
Occupation	
Unemployed	70 (25.5)
Self‐employed	159 (57.8)
Salaried worker	46 (16.7)

### 3.2 Clinical variables of neonates

Out of the 275 neonates clinically diagnosed with sepsis, 55.6% (153/275) were males, and 44.6% (122/275) were females, giving a male‐to‐female ratio of 1.25:1. The majority, 79.3% (218/275) of these neonates, were admitted with EOS, with 20.7% (57) admitted with LOS. The majority, 58.7% (128/218), of the neonates admitted with EOS were delivered with normal birth weight, while 66.7% (38/57) of the neonates who presented with LOS were delivered with normal birth weight. Though marginally, low birth weight was significantly associated with EOS *χ* ^2^ (1, *N* = 218), =0.14, *p* = .04). The average gestational age of neonates admitted with EOS (35.4 ± 3.7 days) was significantly higher than those who were admitted with LOS (34.7 ± 5.9 days) (*χ* ^2^ [3, *N* = 218] = 0.02 *p* = .01). Statistically, there was a significant association between the duration of stay at the unit and the type of sepsis, with neonates admitted with LOS (5.9 ± 9.0 days) spending more days than those admitted with EOS (2.9 ± 2.3 days). Neonates admitted with LOS had a higher mean axillary temperature (37.0 ± 1.6°C) than those admitted with EOS (36.1 ± 3.9°C). A significant proportion of neonates with EOS (*χ* ^2^ [3, *N* = 218] = 0.46, *p* < .001) and LOS (*χ* ^2^ [3, *N* = 57] = 2.82, *p* < .000) were born at the TTH. The mean number of conditions clinically diagnosed for all the neonates was 1.5 ± 0.8. Neonates who were admitted with EOS had a statistically significantly lower number of clinical diagnoses conditions (*χ* ^2^ [3, *N* = 218] = 1.27, *p* < .000) compared with neonates with LOS (Table 2).

**Table 2:** Clinical variables of neonates stratified by the onset of sepsis.

Variable	Total (*N* = 275)	EOS (*N* = 218)	*χ* ^2^, *df*	*p*‐value	LOS (*N* = 57)	*χ* ^2^, *df*	*p‐v* alue
Age on admission (days)	*n* (%)	*n* (%)			*n* (%)		
Mean ± SD	5.6 ± 7.4	2.8 ± 1.7			16.3 ± 10.6		
Sex							
Male	153 (55.6)	118 (54.1)	0.12−1	.22	35 (61.4)	0.22−1	.09
Female	122 (44.4)	100 (45.9)			22 (38.6)		
Birth weight (kg)							
Mean ± SD	2.6 ± 0.8	2.5 ± 0.8			2.7 ± 0.7		
LBW < 2.5	109 (39.6)	90 (41.3)	0.14−1	.04	19 (33.3)	0.19−1	.01
NBW ≥ 2.5	166 (60.4)	128 (58.7)			38 (66.7)		
Gestational age (weeks)							
Mean ± SD	35.3 ± 4.2	35.4 ± 3.7			34.7 ± 5.9		
Preterm	161 (58.6)	128 (58.7)	0.02−1	.01	33 (57.9)	0.01−1	.23
Term	114 (41.4)	90 (41.3)			24 (42.1)		
Duration of hospital stay (days)							
Mean ± SD	3.6 ± 4.8	2.9 ± 2.3			5.9 ± 9.0		
1–3	184 (66.9)	152 (69.7)	1.24−2	<.000	32 (56.1)	9.48−2	.001
4–7	69 (25.1)	54 (24.8)			15 (26.3)		
>7	22 (8.0)	12 (5.5)			10 (17.6)		
Temperature on admission (°C)							
Mean ± SD	36.4 ± 2.8	36.1 ± 3.9			37.0 ± 1.6		
Hypothermia	98 (35.6)	82 (37.6)	0.61−2	.001	16 (28.1)	5.06−2	.69
Normothermia	111 (40.4)	90 (41.3)			21 (36.8)		
Hyperthermia	66 (24.0)	46 (21.1)			20 (35.1)		
Place of delivery							
TTH	201 (73.1)	156 (71.6)	0.46−3	<.0001	45 (79.0)	2.82−3	<.000
2° facility	44 (16.0)	34 (15.6)			10 (17.5)		
Clinic	24 (8.7)	22 (10.1)			2 (3.5)		
Home	6 (2.2)	6 (2.7)			0 (0.0)		
No. of diagnosis per neonate							
Mean ± SD	1.7 ± 0.8	1.6 ± 0.8			1.9 ± 0.9		
1	147 (53.5)	124 (56.9)	1.27−3	<.000	23 (40.4)	9.34−3	.50
2	74 (26.9)	58 (26.6)			16 (28.1)		
3	50 (18.2)	32 (14.7)			18 (31.5)		
4	4 (1.5)	4 (1.8)			0 (0.0)		

Abbreviations: EOS, early‐onset of sepsis; LOS, late‐onset of sepsis; LBW, low birth weight; NBW, normal birth weight; TTH, Tamale Teaching Hospital; °C, degree Celsius.

### 3.3 Predictors of the onset of sepsis among neonates admitted to the NICU, TTH

Presented in Table 3 is a univariate and multivariate logistic regression analysis to determine maternal sociodemographic characteristics and neonatal clinical variables as predictors of EOS. Neonates delivered by single mothers were approximately two times more likely to present with EOS (AOR = 1.882, 95% CI = 0.926−3.822, *p* = .08), with neonates delivered at home being approximately four times more likely (AOR = 3.667, 95% CI = 0.584−23.026, *p* = .17) to be admitted with EOS though this was not stat significant. When maternal occupation was adjusted for in a multivariate analysis, neonates delivered by single mothers were twice as likelier to be admitted with EOS, although marginally significant (AOR = 2.167, 95% CI = 1.010−4.648, *p* = .04). Other maternal sociodemographic characteristics and neonatal clinical variables were not significantly associated with EOS (Table 3).

**Table 3:** Univariate and multivariate logistic regression analysis of maternal and neonatal predictors of early‐onset of sepsis among neonates admitted to the NICU, TTH.

Variable	Univariate		Multivariate	
	OR (95% CI)	*p*‐value	AOR (95% CI)	*p*‐value
Maternal age (years)				
<20	0.625 (0.222−1.757)	.37	0.622 (0.221−1.749)	.37
20−29	1.250 (0.674−2.320)	.48	1.248 (0.672−2.317)	.48
>29	1		1	
Marital status				
Single	1.882 (0.926−3.822)	.08	2.167 (1.010−4.648)	.04
Married	1		1	
Educational status				
None	0.732 (0.395−1.358)	.32	0.733 (0.390−1.379)	.34
Basic	1.211 (0.588−2.493)	.60	1.211 (0.588−2.493)	.60
≥Secondary	1		1	
Occupation				
Unemployed	0.892 (0.483−1.646)	.71		
Employed	1			
Neonatal birth weight				
LBW	0.607 (0.351−1.051)	.08	0.610 (0.352−1.057)	.08
NBW	1		1	
Gestational age of neonate				
Preterm	0.634 (0.364−1.105)	.12	0.638 (0.364−1.117)	.12
Term	1		1	
Place of delivery of neonate				
Home	3.667 (0.584−23.026)	.17	3.673 (0.577−23.374)	.17
Clinic	1.742 (0.806−3.763)	.16	1.743 (0.804−3.779)	.16
Secondary	0.688 (0.213−2.221)	.53	0.687 (0.213−2.221)	.53
TTH	1		1	

Abbreviations: LBW, low birth weight; NBW, normal birth weight; TTH, Tamale Teaching Hospital.

Presented in Table 4 are the univariate and multivariate logistic regression to predict maternal sociodemographic characteristics and neonatal clinical variables for LOS. In the univariate analysis, neonates delivered by single mothers were approximately three times more likely to be admitted to the NICU with LOS. However, this was not statistically significant (AOR = 2.906, 95% CI = 0.715−11.805, *p* = .14). Unemployed mothers, though marginally significant, were less likely to be associated with LOS (AOR = 0.192, 95% CI = 0.038−0.961, *p* = .04). When mothers' occupational status was adjusted for in a multivariate regression analysis, low birth weight, though significant, was less likely to be associated with LOS (AOR = 0.193, 95% CI = 0.038−0.971, *p* = .04).

**Table 4:** Univariate and multivariate logistic regression analysis of maternal and neonatal predictors of late‐onset sepsis among neonates admitted to the NICU, TTH.

Variable	Univariate		Multivariate	
	OR (95% CI)	*p‐v*alue	AOR (95% CI)	*p‐v*alue
Maternal age (years)				
<20	0.500 (0.070−3.550)	.49	1.228 (0.125−12.056)	.86
20−29	0.522 (0.157−1.738)	.29	0.582 (0.167−2.023)	.40
>29	1		1	
Marital status				
Single	2.906 (0.715−11.805)	.14	1.002 (0.129−3.765)	.45
Married	1		1	
Educational status				
None	1.000 (0.232−4.310)	.99	1.460 (0.302−7.070)	.64
Basic	0.818 (0.164−4.091)	.81	0.607 (0.115−3.207)	.56
≥Secondary	1		1	
Occupation				
Unemployed	0.192 (0.038−0.961)	.04		
Employed	1			
Neonatal birth weight				
LBW < 2.5	1.247 (0.405−3.837)	.70	0.193 (0.038−0.971)	.04
NBW 2.5 above	1		1	
Gestational age of neonate				
Preterm	1.095 (0.359−3.338)	.87	1.255 (0.387−4.069)	.71
Term	1		1	
Place of delivery of neonate				
Home	NA	NA	NA	NA
Clinic	NA	NA	NA	NA
Secondary	1.000 (0.247−4.050)	.99	1.109 (0.258−4.776)	.89
TTH	1		1	

Abbreviations: LBW, low birth weight; NA, not applicable; NBW, normal birth weight; TTH, Tamale Teaching Hospital.

## 4 DISCUSSION

Neonatal sepsis is a medical emergency. Identifying factors predisposing neonates to this health problem can reduce the disease burden on Ghana's already constrained health care system. This study is the first prospective study to determine predictors of neonatal sepsis at the TTH, with the only previous study being retrospective.^25^ Clinical signs of neonatal sepsis are usually nonspecific, and neonatologists/pediatricians have a high index of suspicion for prompt diagnosis to avert possible mortality and morbidity in the newborn.

In this study, the marital status of mothers, lower gestational age and low birth weight were the main predictors of the onset of neonatal sepsis. These will provide a valuable local reference for the early identification of neonatal sepsis. In this light, this research was conducted to determine predictors of neonatal sepsis at the NICU of the TTH.

Conflicting results have been reported in the literature regarding the influence of maternal age on neonatal admissions to NICUs. In Ghana, Siakwa et al.^26^ and in Tanzania, Jabiri et al.^27^ reported maternal age's association with neonates' admission into NICUs in their respective countries. On the contrary, this study did not find any influence of maternal age on neonatal admissions into NICUs in Ghana, which is consistent with earlier reports by Aku et al.^28^ and Adatara et al.^29^ It must be noted that the mean maternal age reported in this study was within the range (28.0 ± 6.0 years) as earlier reported among a similar cohort of Ghanaian women by Aku et al.^28^ Also, findings from this study, in which the majority of the mothers were within the age range of 20−29 years, was consistent with the earlier report by Adatara et al.^29^ and the Ghana Demographic Health Survey, which reported the reproductive age of Ghanaian women to be 20−35 years.^30^

The preponderance of male neonates admitted to the NICU reported by previous studies was corroborated in this study.^29^, ^31^ The male neonate is associated with a higher risk of infectious, cardiovascular, pulmonary, and neurological morbidities relative to female neonates. As a result, it is more likely to be admitted to the NICU.^32^, ^33^

The association of gestational age with the incidence of EOS noted in this study was congruent with findings by Mukhopadhyay and Puopolo^34^ and Shane et al.^35^ Low gestational age has been strongly linked with the neonate's immune apparatus's poor development, maturation, and a reduction in pathogen‐specific antibodies acquired from the mother.^36^

It is worth noting that neonates delivered at home were at a higher risk of developing sepsis than those born at a health facility, congruent with reports by Shobowale et al.^3^ This is not surprising as most extramural deliveries, especially home deliveries, are carried out in an environment associated with various predisposing factors, such as unclean and unsafe settings and inadequate staffing, increasing the likelihood of infections.

In this study, the mean duration of stay on admission was lower than the earlier mean duration of stay reported by Vaniya et al.^37^ Other authors have reported a mean duration of stay as high as 22 days.^38^ Neonatal sepsis is considered a clinical emergency, with its management implying the neonate's lifelong survival. Due to this, much of the treatment tends to be completed in the inpatient setting before discharge, necessitating a prolonged duration of admission. However, such a longer duration of stay on admission may come with excess expenditure on the scanty resources and time of immediate family members and the health care delivery system as a whole.

The longer stay at NICUs reported by Bulkowstein, Ben‐Shimol^39^ to be associated with EOS compared with LOS could not be confirmed in this study. However, findings from this study indicated a relatively longer duration of stay for neonates admitted with LOS (5.9 ± 9.0 days) compared to those admitted with EOS (2.9 ± 2.3 days). LOS is more prevalent, possibly due to innumerable high‐risk septic factors during the early phase of an infant's life.

## 5 CONCLUSIONS

This study allows us to conclude that single motherhood is the main predictor of EOS. Low birth weight though not significant, and unemployed mothers are the main predictors of LOS. The findings from this study can serve as a commencement point for developing predictive models for identifying predictors of EOS and LOS in neonates.

## AUTHOR CONTRIBUTIONS

**Kwame Opare‐Asamoah**: Conceptualization; project administration; supervision; writing—original draft. **Samuel E Acquah**: Data curation; writing—original draft. **Ezekial Kofi Vicar**: Formal analysis; methodology; writing—review and editing. **Lawrence Quaye**: Conceptualization; data curation. **Abdul‐Mumin Alhassan**: Data curation. **Saeed F Majeed**: Writing—review and editing. **Vida Nyagre Yakong**: Conceptualization; writing—review and editing. **Samuel Yankson**: Data curation; writing—review and editing. All authors have read and approved the final version of the manuscript.

## CONFLICT OF INTEREST STATEMENT

The authors declare no conflict of interest.

## ETHICS STATEMENT

The ethical approval for the study was obtained from the Ethical Review Committee (ERC) of the Tamale Teaching Hospital, Tamale, Ghana (TTHERC/25/06/19/03). A thumb‐printed consent to participate was obtained from all mothers/caregivers of neonates.

## TRANSPARENCY STATEMENT

The lead author Kwame Opare‐Asamoah affirms that this manuscript is an honest, accurate, and transparent account of the study being reported; that no important aspects of the study have been omitted; and that any discrepancies from the study as planned (and, if relevant, registered) have been explained.

## Data Availability

The data set and the analysis for this work are available from the authors at a reasonable request. Dr. Kwame Opare‐Asamoah had full access to all of the data in this study and takes complete responsibility for the integrity of the data and the accuracy of the data analysis.
